# Notes and News

**Published:** 1888-02-25

**Authors:** 


### NOTES AND NEWS.

COLLECTED AND COMMUNICATED.

HER Majesty the Queen paid an informal visit to the Royal National Hospital for Consumption, Ventnor, on the 11th inst.

THE Jubilee collections received by the Central Committee of the Countess of Dufferin's Fund, between October 16th and December 31st, 1887, amount to Rs. 9,331-0-7.

PRINCE JEHAN KUDR MIRZA MAHOMED WAHED ALI BAHADOOR OF OUDH, of Garden Reach, has sent a donation to the Lady Dufferin's Zenana Hospital, Circular Road, Calcutta, and writes that he hopes the donation will be accepted, "to mark the occasion of her Excellency's visit to an institution which he considers one of the greatest boons conferred on the women of Calcutta."

THE committee of the St. Alban's Hospital anticipate that the new hospital, which is being built on the Verulam Road, will be open on the 1st May.

THE Essex and Colchester Hospital has, in token of their appreciation of Dr. Symmons's services as a surgeon to the hospital during the last nineteen years, offered him the post of consulting surgeon to the institution.

M. WADDINGTON presided at the twentieth annual dinner in aid of the funds of the French Hospital, held at Willis's Rooms on the 11th inst. There were present about 200 supporters of the charity, and at the close of the evening the treasurer announced that subscriptions to the amount of £2,000 had been received.

AT the last meeting of the Hartlepools Hospital a minute was entered gratefully acknowledging the ungrudging services rendered to the hospital by Dr. Rutherford Morison, who has resigned his appointment as honorary physician to the hospital owing to his removal from the town. Dr. Morison is well known as one of the most talented medical men in the north of England, and his resignation is a distinct loss to the Hartlepools Hospital.

PROFESSOR STRUTHERS, writing to the *Aberdeen Journal* about the approaching election of infirmary directors, asks how it comes that no ladies have been nominated for this office. They are now eligible for it, and Dr. Struthers thinks it would be a most suitable and useful arrangement to have two lady members on each board. There are indeed many points in hospital management which are peculiarly woman's work; and we are quite sure that ladies would succeed as well as active directors of hospitals as they do as members of boards of guardians, in which capacity they are now con-considered essential wherever their usefulness has been tested.

THE NATIONAL PENSION FUND is growing in interest and importance. The £20,000 promised by Messrs. Rothschild, Gibbs, Hambro, and J. S. Morgan has been formally deposited in the Court of Chancery, and other large amounts have been paid to the treasurer on behalf of the bonus fund. Already there is a large assured annual income accruing on sums given and placed at interest, available for pensions as they may become due.

THE late Dr. Woollett, for forty years senior honorary medical officer of the Newport Infirmary and Dispensary, has bequeathed by his will £500, free of legacy duty, to the institution he served so long and faithfully. The number of in-patients in the infirmary during last year was 403, and among these only ten deaths occurred, two of which were accident cases. This low mortality proves that Newport Infirmary can boast of both good medical attendance and good sanitation.

AT the annual meeting of the National Children's Hospital, Dublin, Dr. W. Moore said that it was a mistake to suppose that the nursing of children was identical with the nursing in a general hospital. The word gentleness had a meaning of much greater importance in a children's hospital, and even more tact and patience were required in the nurses and medical attendants there than in a general hospital. The annual report of this hospital showed that whereas at the beginning of last year it was £2,000 in debt, it now owed only £1,300.

AT the annual meeting of the Sunderland and North Durham Eye Infirmary Dr. Percy Blumer stated that, so long as the rule remained in force that in-patients at the infirmary must provide their own food, the medical officers could not advise all patients to take advantage of in-door treatment. It is to be hoped, for many reasons, that this rule will soon be abrogated—among others, for the prosperity of the hospital. Hospitals where everything is free are always those most liberally supported by the public, as the Newcastle people are finding out.

AT the annual meeting of the subscribers to the Royal Portsmouth, Portsea, and Gosport Hospital, which was held in the Guildhall, Portsmouth, on the 14th inst., there were many allusions to the enlargement of the hospital at present going on. Dr. J. Ward Cousins stated that at present they were in a "magnificent mess" at the hospital, but that after it was over they hoped to have accommodation for 100 beds, and instead of obsolete, ill-ventilated wards, they would have good, convenient wards, with every modern appliance. The Jubilee Fund, raised by the late Mayor, Sir William King, is not to be touched for any reason whatever, and the interest on it will give the hospital a permanent income of £130 per annum. The Admiralty wards, which had been closed, have now been re-opened to a limited extent.

THE fourteenth annual meeting of the Hospital Saturday Fund was held at the Memorial Hall, Farringdon-street, on the 9th inst., Lord Brassey (president of the association) being in the chair. His Lordship stated that when the collections began, the sum produced was £250, gained by the efforts of 24 lady collectors. Last year the fund amounted to nearly £10,000. Mr. S. C. Bartley, M.P., mentioned in the course of a short speech that it was calculated that a halfpenny a-week collected for six months from the men in the workshops of London would give the fund no less than £50,000. Lieutenant-Colonel Mercier moved a resolution "to extend the work of Hospital Saturday throughout the shops and warehouses of London"; and Alderman Gould, of Kingston-on-Thames, proposed one suggesting the advisability of a conference of local charities, with a view to getting working-men representatives on the boards of management. Both resolutions were carried.

THE following vacancies are announced this week, the amount of salary in each case being given after the name of the institution :—

**Resident Medical Officers.**

Birmingham General Hospital. Assistant House Surgeon.—Board and washing, but no salary
Earlswood Asylum for Idiots, Redhill, Surrey. Medical Superintendent.—£500, with furnished apartments.
French Hospital and Dispensary, Leicester Square, W.C.—£60 per annum, with board and attendance.
Lincolnshire County Asylum, Bracebridge, near Lincoln. Assistant Medical Officer—£150 per annum, with board.
Liverpool Northern Hospital.—£70 per annum, with board.
Manchester Hospital for Consumption and Diseases of the Chest. £50 per annum, with board.
Metropolitan Hospital, Kingsland Road, E. Junior House Surgeon.—£40 per annum, with board.
West Bromwich District Hospital. House Surgeon.—£80 per annum, with board.
York County Asylum Senior House Surgeon.—£100 per annum, with board.
York Dispensary. Three Resident Medical Officers.—£130 per annum.

**Non-resident Medical Officers.**

Brompton Cancer Hospital, S.W. Pathologist.—Honorarium of £80, to be voted at the end of the year.
Central London Orthopædic Hospital, Gray's Inn Road, W.C.—Assistant Surgeon.
Cirencester Union Medical Officer for the Union Workhouse and the South District.—£115 for the joint appointment, with fees
St George's and St James's Dispensary Physician.

**Nurses.**

Devon and Exeter Hospital. One, Trained Surgical.
Children's Hospital, Cardington, near Bedford. One.
Mr. E. D. Wilson's Institution, Wimpole Street. Several, for Female and Male.
Teddington and Hampton Cottage Hospital. Assistant Nurse.—£18, board, lodging, and washing.
Kent Nursing Institution, West Malling One, Hospital Trained.
Workhouse Infirmary Association, 6, Adam Street, Strand. Several.

**Probationers.**

Kent Nursing Institution, West Malling. Several.
Ancoat's Hospital, Manchester. One.

---

MR. JOHN THOMPSON, a working man, writes to the *Manchester Guardian* suggesting that collecting-boxes should be placed in the various schools of the city in connexion with the Hospital Saturday Fund. "Little Tommy and Joey, or Polly and Edie," he says, "if it were pointed out to them that a hospital exists for little children, would readily give their halfpenny, received from father or Uncle Jim, and rejoice in so doing." The suggestion is worth considering all over the country, for children cannot be taught too early the duty of showing kindness to those less fortunately placed than themselves.

---

WE have received the *Quarterly Paper* of the Edinburgh Medical Missionary Society, which contains a gratifying account of the Society's work. It is obvious to every one that medical missions are the most efficacious and successful that can be sent to heathen countries. Pain and disease make all men forget distinctions of creed; and the missionary who would meet with insult, or worse, if his only work were to preach to those he went among a creed different from that in which they had been brought up, wins his way to their hearts and souls if he can show that he possesses the will and power to cure them in illness. Medical missionaries may claim to be in every sense the followers of the Great Physician who went about doing good to both soul and body; and it is to be regretted that the Society is unable to increase the number it sends out. At the last meeting of the Society, Professor Alexander Simpson was appointed president, and Dr. Halliday Douglas vice-president—appointments which must meet with the approval of all who know these gentlemen.

THE Calverley and District Joint Hospital has been built and is maintained by the township whose name it bears, and by those of Eccleshill, Idle, Farsley, and Padsey. All these four are larger than Calverley, and the committee has very sensibly agreed to avoid contention as to the honour of giving the name to the new hospital by conferring it on the member which was outside the rivalry.

---

"OBSERVER," writing to the *Leicester Post*, complains of the fashion in which dispensing is managed at the Provident Dispensary. He visited one of the branches of this institution lately, and was there informed by the lad of seventeen who was in charge, that to send out 160 bottles in two hours was a common occurrence. Quick work this; but is it accurate, as the sending out of medicine ought to be? "What do you do if you are short of a drug?" asked "Observer." "Oh! put something else in," was the candid reply. It is to be hoped that this case is exceptional, and that more care is exercised at the other branches of the dispensary. Nevertheless, the inquiry into the management which has lately been demanded seems to be not altogether unnecessary.

---

THE Lower Sydenham Home and Infirmary for Sick Children has had some cause for anxiety in the diversion of money to other objects owing to the Jubilee festivities, and in the recent epidemic of scarlet fever; but it has not suffered in any marked degree. The balance-sheet shows that the receipts for the year amounted to £1,472 6*s*. 9*d*., and the expenditure to £1,381 10*s*. 3*d*., which leaves a balance of £90 16*s* 6*d*. to the credit of the institution. Very few patients suffered from scarlatina, and these were slight cases; but, nevertheless, fresh cases had to be excluded for a time, and finally the home had to be closed for a few weeks for disinfecting and cleansing. Owing to this the number of patients treated during last year was less than in 1886, being 481 as against 549.

---

A NEW doctor has offered his services to the plague-stricken town of Sheffield, and one of unique quality. Mr. Herring, of Leeds, undertakes to cure sufferers from small-pox at the rate of exactly thirty per diem. The method he employs is not confided to the public, who seem, nevertheless, to be sufficiently ready to take it on trust. Two thousand of the inhabitants of Sheffield held a public meeting, in which they called upon the Mayor, as chairman of the Board of Health, to adopt the new method—whatever it is—and received his refusal to entrust the lives of the citizens to the miracle-doctor with every mark of disfavour. The meeting must have been interesting and exceptionally representative of the population, for we are told that "many of those present were suffering from small-pox." Nevertheless, we feel a mild thankfulness that we were not among them.

---

THE Church of England Central Society for Providing Homes for Waifs and Strays is doing a most useful work. Its object is to remove children from vicious surroundings and place them where they can grow up into honest and useful citizens. The short life histories given of some of the children whom the Society have rescued show how much such rescue was needed; but it may be hoped that, being taken from the brutal and unprincipled parents and guardians who neglected and ill-used them—except when they could profit by their earnings—they may forget the evil example that was formerly set before them. The youngest children adopted by the Society are boarded out in families, others are kept in small homes established by the Society, and the eldest are emigrated to Canada. During last year the number of children received by the Society was 377, or rather more than one a day.

